# The conduct and reporting of qualitative evidence syntheses in health and social care guidelines: a content analysis

**DOI:** 10.1186/s12874-022-01743-1

**Published:** 2022-10-12

**Authors:** Chris Carmona, Susan Baxter, Christopher Carroll

**Affiliations:** School of Health and Related Research (ScHARR), University of Sheffield, Sheffield, UK

**Keywords:** Qualitative evidence synthesis, Reporting frameworks, Guideline development

## Abstract

**Background:**

This paper is part of a broader investigation into the ways in which health and social care guideline producers are using qualitative evidence syntheses (QESs) alongside more established methods of guideline development such as systematic reviews and meta-analyses of quantitative data. This study is a content analysis of QESs produced over a 5-year period by a leading provider of guidelines for the National Health Service in the UK (the National Institute for Health and Care Excellence) to explore how closely they match a reporting framework for QES.

**Methods:**

Guidelines published or updated between Jan 2015 and Dec 2019 were identified via searches of the National Institute for Health and Care excellence (NICE) website. These guidelines were searched to identify any QES conducted during the development of the guideline. Data relating to the compliance of these syntheses against a reporting framework for QES (ENTREQ) were extracted and compiled, and descriptive statistics used to provide an analysis of the of QES conduct, reporting and use by this major international guideline producer.

**Results:**

QES contributed, in part, to 54 out of a total of 192 guidelines over the five-year period. Although methods for producing and reporting QES have changed substantially over the past decade, this study found that there has been little change in the number or quality of NICE QESs over time. The largest predictor of quality was the centre or team which undertook the synthesis. Analysis indicated that elements of review methods which were similar to those used in quantitative systematic reviews tended to be carried out well and mostly matched the criteria in the reporting framework, but review methods which were more specific to a QES tended to be carried out less well, with fewer examples of criteria in the reporting framework being achieved.

**Conclusion:**

The study suggests that use, conduct and reporting of optimal QES methods requires development, as over time the quality of reporting of QES both overall, and by specific centres, has not improved in spite of clearer reporting frameworks and important methodological developments. Further staff training in QES methods may be helpful for reviewers who are more familiar with conventional forms of systematic review if the highest standards of QES are to be achieved. There seems potential for greater use of evidence from qualitative research during guideline development.

**Supplementary Information:**

The online version contains supplementary material available at 10.1186/s12874-022-01743-1.

## Introduction

Evidence-based health and social care guidelines (including clinical, public health and social care guidelines) are part of the landscape of evidence-based health and social care in many countries. These guidelines are normally based on one or more analyses of relevant evidence, often in the form of systematic reviews of effectiveness data, and often interpreted by an expert committee.

Even though methods for synthesising qualitative research have been around for many years, interest in the use of qualitative evidence to inform the development of these guidelines has grown considerably over recent years. This is partly because of key developments such as more robust methods of synthesis, development of tools like GRADE CERQual and better frameworks for reporting qualitative studies [1] and partly because qualitative data can answer particular types of questions better than quantitative data. Quantitative data are still key for questions of efficacy, but are less able to answer questions relating to the effects of patient preference, feasibility and acceptability on the broader effectiveness of a treatment or intervention. These questions are best answered by qualitative studies. [2].

The World Health Organization (WHO) handbook [3] affirms that qualitative evidence should be used in the process of guideline development, and the Cochrane Qualitative and Implementation Methods group are planning to publish a manual for qualitative evidence synthesis in 2023. Other leading international guideline producers, such as the UK National Institute for Health and Care Excellence (NICE) are using qualitative evidence syntheses, both alone and as part of mixed methods reviews, to present evidence to their guideline committees and this is supported by initiatives such as GRADE CERQual [4] that have been developed with guideline committees specifically in mind. This surge of interest led Lewin and Glenton to declare “a new era” for qualitative research [1]. A recent paper exploring how developers use qualitative evidence searched internationally for guidelines that used qualitative research and appraised their quality [5]. The authors rated the guidelines using the AGREE II criteria, finding that most of the guidelines were of high quality. However, the AGREE criteria are intended to assess the methodological quality of the guideline itself and the authors did not investigate the reporting of the evidence reviews that informed the guideline.

A short paper published by Tan and colleagues in 2009 [10] explored the use of qualitative evidence by NICE between 2002 (when NICE produced its first guidelines) and 2007. The authors reported that almost 50% of NICE guidelines produced in that period made use of qualitative studies, although they did not report whether those are single qualitative studies or whether any qualitative evidence synthesis was undertaken. The paper noted a growing trend by year in terms of the numbers of qualitative studies used in guidelines, rising from nine studies in 2003 to 41 in 2004, 60 in 2005 and 139 studies in 2006. The authors attributed the growth in the number of qualitative studies used to a combination of two factors. Firstly, a shift toward producing more guidelines on chronic conditions, where they argued that patient needs constituted an important part of the guideline, and secondly, that NICE’s developing policy emphasis on patient and carer involvement led to more attention being paid to patient and carer perspectives.

They further noted that only five of the 22 guidelines which drew on qualitative research used (or documented) specific search strategies for qualitative literature over and above searches that were done for quantitative studies. Only four of the guidelines documented key methodological process details such as inclusion/exclusion criteria for qualitative studies.

This study also highlighted a gap in the reporting of the reviews - only half (11/22) of the guidelines reported how critical appraisal of qualitative studies was carried out, and only three of the 22 reported how data were synthesised.

The study concluded that “there is no consistency in how qualitative evidence is utilised in the development of NICE clinical guidelines. There are also clear training needs for NICE’s guideline developers in terms of how best to identify, quality appraise and synthesise qualitative evidence” (p.172).

The work reported in this current paper updates the study by Tan and colleagues by exploring whether methodological changes within NICE, or development in methodological standards for QES have led to a change in their use in NICE guidelines. It also builds on a review of methodological literature by the current authors [6]. The study aims to examine all qualitative evidence syntheses used in guideline documents published between 2015 and the end of 2019 by a leading producer of guidelines for clinical, public health, and social care in the UK. NICE was chosen as an appropriate exemplar because of its international reputation as a leading guideline producer. The study aimed to explore where and how QES are used in the development of health and social care guidelines, and how the methodologies used compare with international standards of good practice.

## Method

The study used a content analysis method to analyse textual data. [7] Berelson described content analysis as “a research technique for the objective, systematic and quantitative description of the manifest content of communication” (p. 18). [8] Content analysis incorporates both quantitative approaches that convert the textual data to numerical data, for example by counting occurrences of the content of interest, and also more qualitative approaches that analyse the way that the content of interest in presented or discussed. The process followed in this study was based on the method outlined by Bengtsson (see Table 1). [9].

**Table 1:** Summary of Bengtsson method for content analysis

Stage	Tasks	How was this operationalised?
**Planning**	• Aim • Sample & unit of analysis • Data collection • Method of analysis • Practical implications	• Aim – to better understand variation in the reporting of QES used in NICE guidelines • Sample – NICE guidelines published or updated 2015–2019 • Unit of analysis – A single QES was the unit of analysis rather than the guideline as a whole since some guidelines have multiple associated QES • Data collection/analysis – see boxes below • Practical implications – understanding where QES in the sample do not meet the criteria set out by ENTREQ is a useful indicator of reporting quality.
**Data collection**	• Collect data and transform to analysable text	• Overall set of eligible guidelines identified using functionality on NICE website. • Manual sifting of reviews undertaken for guidelines to identify QES • QES downloaded as pdf documents for analysis.
**Data analysing**	• Categorisation • Compilation	• ENTREQ reporting criteria used as framework for categorisation with each element assessed as ‘met’ or ‘not met’ • Compiled in tabular form in spreadsheet.
**Reporting**	• Creating a report/presentation of the result.	The results are presented in this paper

### Source documents

In order to compare recent NICE guidelines with the sample included by Tan et al. [10], and to reflect current practice, we scrutinised guidelines from a 5-year period (the beginning of 2015 until the end of 2019).

Using inbuilt functionality on the NICE website, a search was conducted for guidelines published between January 2015 and December 2019. This search encompassed the three types of evidence-based guideline produced by the guideline development centres at NICE, classified on the website as ‘public health’, ‘social care’ or ‘clinical’. It does not include guidelines where the method of development differed, that is, antimicrobial guidelines, cancer service guidelines, COVID-19 guidelines and medicines practice guidelines (less than 40 guidelines in total). The resulting list of guidelines was copied to the clipboard (using the website functionality) and pasted into an excel spreadsheet (Microsoft Office Professional Plus 2019).

For each included guideline, the individual evidence reviews (systematic reviews and qualitative evidence syntheses) were explored using the ‘evidence’ tab on the guideline webpage.

Each evidence review was examined to evaluate whether or not a qualitative evidence synthesis (defined as 2 or more qualitative studies combined together to answer the same review question) had been undertaken by the technical team (or a contractor) responsible for the development of the guideline. Evidence reviews that did not report the use of qualitative evidence synthesis (or mixed-methods synthesis with a qualitative component) were excluded from the sample. Any qualitative reviews and mixed methods reviews identified were downloaded and saved. These formed the sample for the content analysis.

### Data collection

Included QES were copied to a new excel spreadsheet and rationalised so that the unit of analysis was the qualitative evidence synthesis rather than the guideline (some guidelines were supported by multiple qualitative evidence syntheses). The coding framework (described below) was added to the spreadsheet to create a data extraction tool.

The coding framework used was intended to provide two sets of data – descriptive data and content data.

### Descriptive data

This included key data from the QES – guideline number, year of publication, author (by guideline producing centre rather than individual authors) and number of qualitative studies included in the analysis. The use of GRADE CERQual [4] to assess the confidence was also noted.

### Content data

The criteria set by ENTREQ [11] are the most commonly used reporting framework for QES, and therefore this framework was selected as a useful one for examining the content of the QES included in this study – see Table 2 and Additional File 1. There are alternative reporting standards for specific types of QES, for example the eMERGe Reporting Guidance for meta-ethnography [12], but since NICE has not produced any of these types of QES they were not used in this analysis.

**Table 2:** Summary of ENTREQ criteria

- Aim - Synthesis methodology - Approach to searching - Inclusion criteria - Data sources - Electronic Search strategy - Study screening methods - Study characteristics - Study selection results - Rationale for appraisal	- Appraisal items - Appraisal process - Appraisal results - Data extraction - Software - Number of reviewers - Coding - Study comparison - Derivation of themes - Quotations - Synthesis output

### Data analysis

Each of the QES was read and descriptive data and content data were coded into an excel spreadsheet according to the framework described above and in Additional file 1. Coding was binary and indicated whether the QES reported on the criterion in the reporting framework or not. For example, did the QES report its aim? Did it report the synthesis methodology it is underpinned by? This approach did not allow for any judgment about the adequacy of each reporting criterion, only whether it was present or not. This approach was taken to allow for analysis of coding.

Resulting data are presented predominantly as descriptive statistics to show trends, consistencies and inconsistencies in the data. Data were visualised using Microsoft Excel or were imported into the R program [13], using the ‘tidyverse’ package [14] to manage the data and the ‘ggplot2’ package [15] (also part of the tidyverse) for data visualisation. The R code used to generate the figures can be found in Additional File 1.

## Results

### Number and size of QES undertaken

Between January 2015 and December 2019, NICE published 192 clinical, public health and social care guidelines. The website categorises the breakdown of these guidelines as 156 clinical, 30 public health and 48 social care guidelines, however this includes some guidelines listed in more than one category, hence the discrepancy in numbers. For the purposes of this analysis, pragmatic decisions were made about the main topic area of a guideline to assign each guideline to a single category, resulting in a breakdown of 143 clinically focussed guidelines, 25 public health focussed guidelines and 24 social care focussed guidelines. Each of these guidelines is based on multiple sources of evidence – most often systematic reviews of quantitative evidence, but also prognostic and diagnostic reviews (of the predictive or diagnostic accuracy of tests or indicators), epidemiological studies (of prevalence and incidence) and, more rarely, qualitative evidence syntheses. The total number of reviews (both quantitative and qualitative) conducted for a guideline can range from one review for an update of a single clinical question to around 40 reviews for a large guideline with multiple questions. The reviews are conducted by expert review teams who present them to the guideline committee. The committee who undertake a structured discussion (although not using a formal evidence to decision framework) of the evidence contained in the reviews (and their confidence in that evidence if GRADE CERQual was used), alongside any other evidence, and contextualise it using their expertise and experience of the UK health and social care system to make guideline recommendations. When a guideline is published, all of the evidence considered by the committee is also published alongside the guideline.

Of the 192 guidelines referred to above, 54 guidelines (28%) had one or more QES as part of their evidence base (qualitative evidence syntheses defined as a synthesis of more than one qualitative study). Overall, out of a total of approximately^1^ 1,500 reviews/research questions, 90 were QES (approx. 6%).

Of the 54 guidelines with one or more QES, 36 (out of a total of 143 [25%]) were clinically focussed, 13 (out of 25 [52%]) were public health focussed, 5 (out of 24 [21%]) were social care focussed. This shows that social care and clinically focussed guidelines are roughly half as likely to use qualitative evidence synthesis as public health focussed guidelines.

The number of QES used per included guideline ranges from 1 to 6 (mean = 1.67 per guideline that contains a QES, less than 0.4 QES per guideline published between Jan 2015 and Dec 2019).

**Fig. 1:**
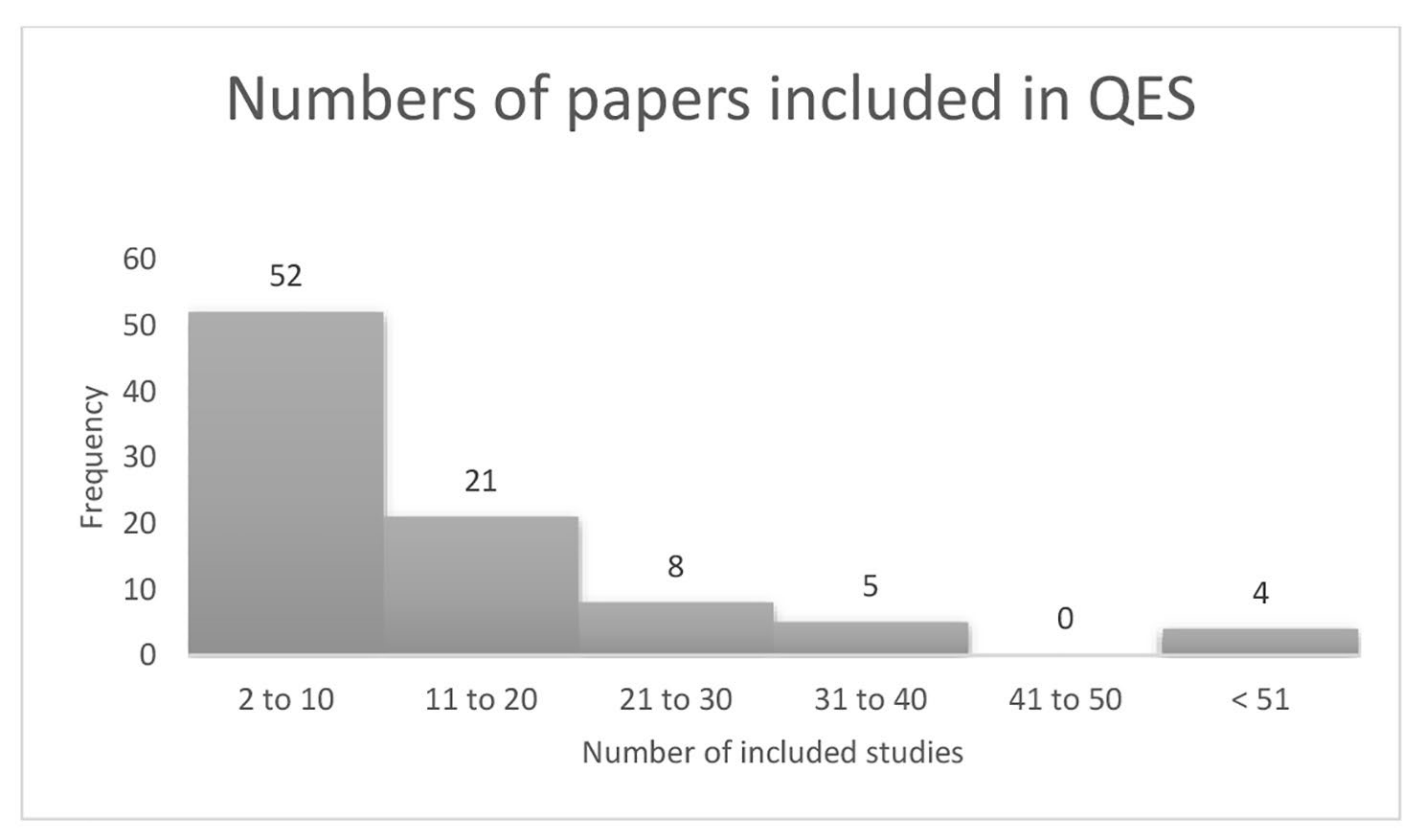
Frequency of QES by number of included papers

In terms of the number of included papers in the QES, there was a large amount of variation. The largest QES contained 69 papers, the smallest QES contained two papers. Distribution of QES by the number of included papers is shown in Fig. 1. Reasons for the variation were not explored as part of this analysis but may be related to the size of the evidence base, or to the formulation of the review protocol.

Overall, 65% (58 out of 90) of QES had less than 12 papers included, with a mode of four and a median of 10 papers. The four QES with more than 42 papers were from two guidelines [16],[17] and in both cases a single set of included papers was identified through searching and sifting and the data were extracted from the single set of papers to develop two QES with different review questions.

**Fig. 2:**
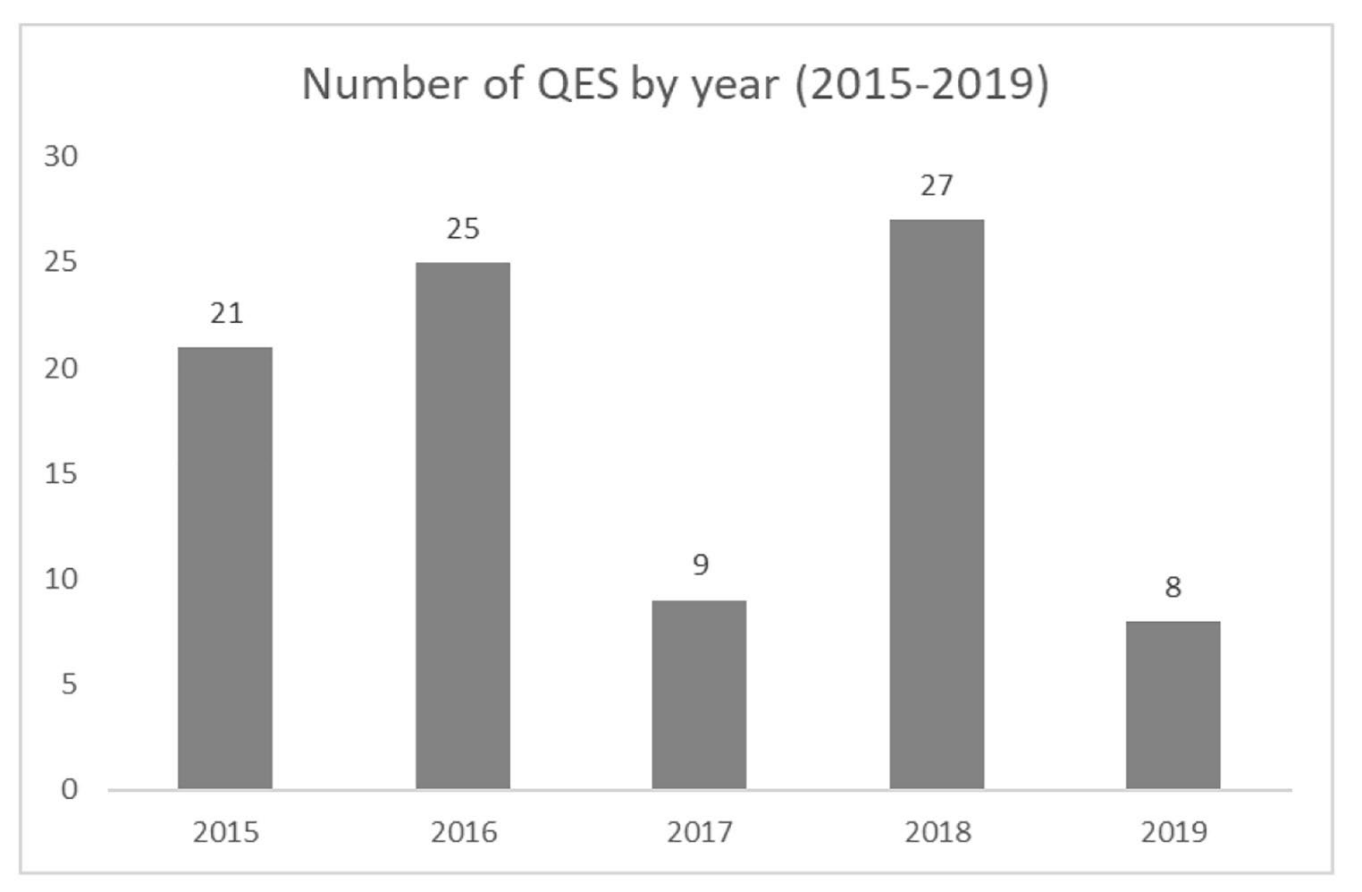
Number of QES published by year (2015–2019)

Figure 2 shows the number of QES conducted by year for the period 2015–2019. The graph does not indicate any meaningful trend toward producing more QES in spite of the growth in acceptability of QES in evidence-based health and social care, and the development of more rigorous methods (see methodological review). The large variations in 2017 and 2019 might be at least partly explained by the lifecycle of a guideline. In most cases guidelines take longer than a year to develop and publish. The number of guidelines published per year is somewhat variable, depending on the length of the guidelines’ development – guidelines with more review questions, usually addressed sequentially, tend to have longer development times. There is no evidence found by this analysis that would indicate why 2017 and 2019 were years when fewer QES were published.

### Purpose of QES undertaken

There are a range of QES methodologies which vary widely on the epistemological spectrum, and in level of complexity, from aggregative approaches to more configurative/interpretive approaches. QES undertaken for NICE guidelines all use simpler descriptive or aggregative approaches. These syntheses can be used to address a range of issues that concern people’s (both patients and healthcare professionals) views, beliefs and lived experiences. While quantitative evidence is best for addressing questions of efficacy (does treatment A have an effect on condition B?), qualitative evidence can be useful to bridge the gap between efficacy and real-life effectiveness, for example understanding why people do not take their medicines as prescribed, how the medicines impact their lives and how things could be improved. In spite of this, guidelines produced by NICE in the period 2015–2019 seem to address a much more limited range of question types using QES. Almost half of the QES undertaken answer one of two types of question:

- What are the barriers and/or facilitators to……?
- What are the information (and support) needs of ……?

Many of the remaining questions deal with similar question types, often about support and care needs. This may indicate a limited understanding in the NICE guideline development centres of the potential remit of QES and their flexibility with regards to issues such as service configuration, professional support etc. Other kinds of QES do include occasional innovative questions, for example one QES for guideline NG77 (management of cataracts in adults) [18] was employed to explore how lens implant errors happen through qualitative analysis of physician reports and case studies.

### Quality of reporting

The 90 QES published by NICE between Jan 2015 and Dec 2019 were assessed against the ENTREQ reporting criteria as described in Table 1 (above) and in more detail in Supplementary Material 1.

**Fig. 3:**
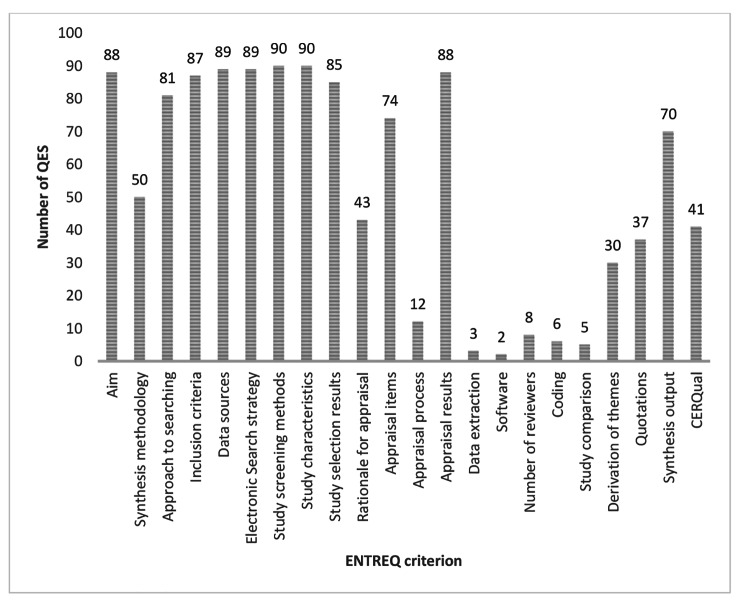
Number of QES (out of 90) meeting each ENTREQ reporting criterion

Analysis of number of guidelines meeting each of the ENTREQ criteria is shown in Fig. 3 with an additional column to indicate whether the QES used GRADE CERQual to assess confidence in the qualitative findings.

ENTREQ criteria relating to setting out the aim of the review and to the systematic searching and sifting of studies to generate a pool of included studies was generally done well and described adequately in the included QES. The exception to this was the synthesis methodology criterion (described by the ENTREQ statement as “Identify the synthesis methodology or theoretical framework which underpins the synthesis, and describe the rationale for choice of methodology”). Many QES (40/90) were marked down on this criterion because either they only provided a brief sentence or statement to describe the methods of data synthesis used, for example “We undertook thematic synthesis”, with no methodological detail, or simply provided inadequate descriptions of methodology, often not specifying an approach to synthesis at all.

Derivation of themes (described by the ENTREQ statement as “Explain whether the process of deriving the themes or constructs was inductive or deductive”) was demonstrated in a third of QES, and these were mostly undertaken by a particular guideline developer who present a ‘theme map’ as a standard part of their QES.

In 70 of the reviews, synthesis output (described by the ENTREQ statement as “Present rich, compelling and useful results that go beyond a summary of the primary studies”) was reported. This was mostly in the form of NICE evidence statements, although some evidence statements made no attempt at synthesis and simply listed the themes identified by individual studies. Some QES used a Cochrane style ‘Summary of qualitative findings’ table to present synthesised themes and sub-themes along with their CERQual confidence rating. Other than that, CERQual was not often used. This does not seem to be dependent on the age of the review (as might be expected given the introduction of CERQual in 2015) but seems to depend more on the guideline developer.

### Variation over time

It might be expected that adherence to reporting frameworks improves over time as methods for undertaking QES become more robust and more widely known. It might also be expected that guideline developers would develop their methods for QES (and train their staff in those methods), and that more recent iterations of the NICE guideline methods manual might give clearer direction on its expectations from QES.

**Fig. 4:**
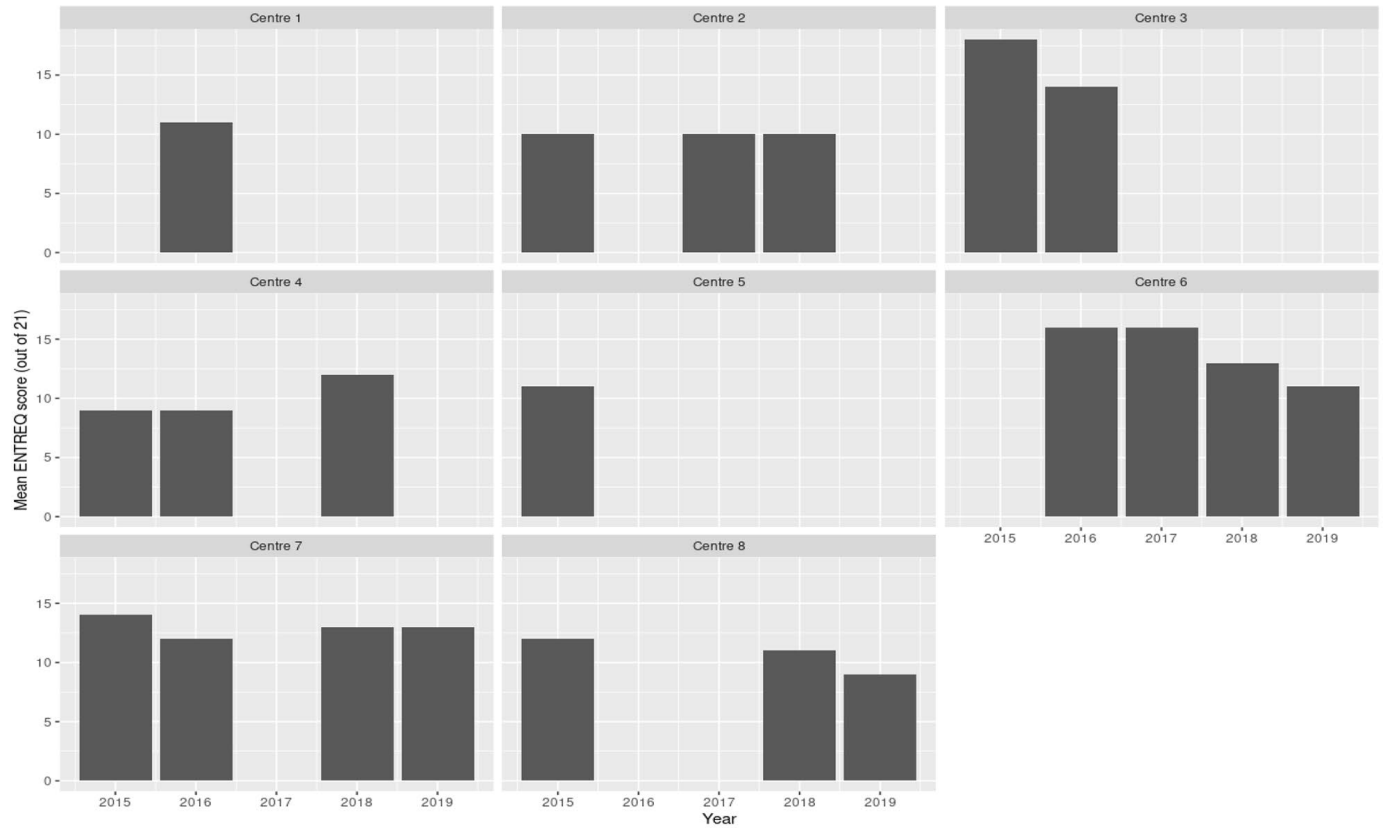
ENTREQ criteria (out of a maximum of 21) reported by year and authoring centre

Figure 4 explores how well QES from different centres match with criteria in the ENTREQ reporting framework over time. For years where a centre produced more than 1 QES, the mean of the number of criteria in the framework (out of 21) for the QES produced in that year is used. It is important to note that using a mean number of reporting criteria is somewhat arbitrary since it requires making a generalisation that each of the 21 criteria in the framework is of equal importance to the reporting of a QES.

Data suggest that in fact there is little variation over time, but that the main determinant of the number of ENTREQ criteria reported is the guideline developer who authored the review. Of the two guideline developers who authored the majority of the QES in the past 5 years, one reasonably consistently reports around 11–13 criteria (Centre 7), whereas the other performs better in 2016 and 2017, but drops to a similar level in 2018 and 2019 (Centre 6). It is unclear what may drive the drop. Two possible confounding factors are the publication of the new NICE methods manual [19] in 2018, or simply a change in staff or senior staff from someone more familiar with QES to someone less familiar.

**Fig. 5:**
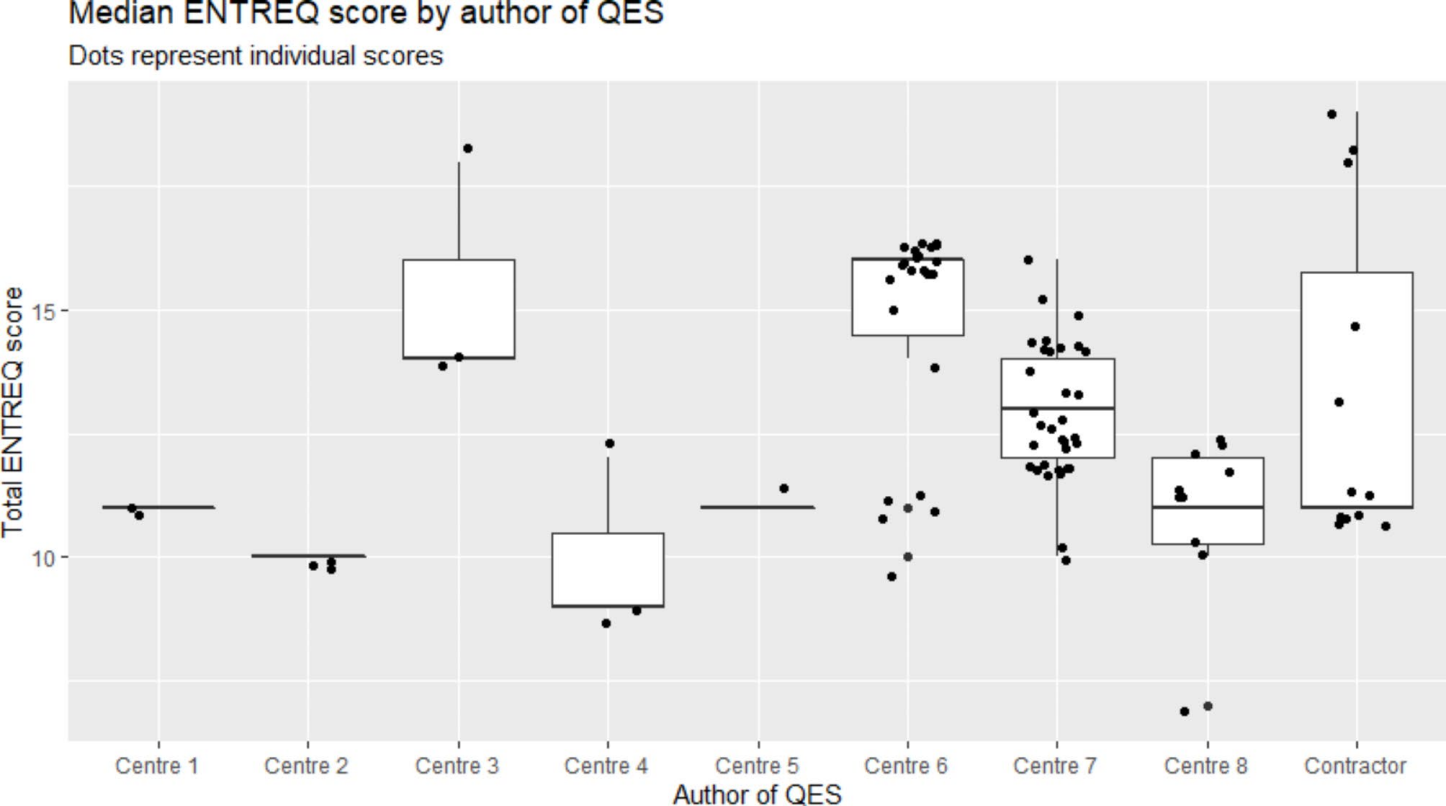
Median number of criteria in the ENTREQ framework met (dots represent individual QES)

To further explore this, data were plotted to calculate the median number of ENTREQ criteria reported over all years (2015–2019) by guideline developer. Figure 5 presents this data along with the associated point values for each QES.

The data in Fig. 5 broadly support the hypothesis that the different producers of QES account for most of the variation in the number of criteria reported on in the reporting framework. Centres that do less well tend to have only produced 2 or 3 QES over the 5 years period and therefore staff are likely to have been less familiar with QES methods having done them rarely. The Centre 8 team do not appear to fit this pattern. Their QES perform poorly against the ENTREQ framework, however the team have produced 11 QES in the 5-year time-frame, including the lowest scoring and second lowest scoring.

The widest variation in meeting the criteria in the framework is seen in the contractor group, but this is to be expected since it is a heterogeneous group comprised of various organisations and academic groups. Since these QES were contracted out, it is reasonable that the highest ranking QES are in this group since competitive tendering would lead to these syntheses being undertaken by specialist teams familiar with QES.

Centres 6 and 7 are the most prolific producers of QES, with centre 7 demonstrating a wide range of reporting quality across their QES. Centre 6 reporting quality appears to be dichotomous with a cluster of QES scoring 10 or 11, and a larger cluster scoring 15 or 16. It is unclear what the cause of this dichotomy might be.

## Discussion

### Number and size of QES undertaken

The number of QES undertaken by NICE (including its contractors) over the 5-year period up to the end of 2019 formed a fraction of the total number of reviews undertaken in the period. Although it is difficult to ascertain why this is the case, there are plausible explanations that can at least partially explain this lack of attention to the qualitative evidence.

The majority of the guidelines produced in the period were clinical guidelines (143 out of 192), and clinical guidelines are most often about the relative efficacy of different treatment modalities. In questions of efficacy, the gold standard is the randomised controlled trial, or a systematic review of randomised controlled trials. Although QES could be used to bridge the efficacy – effectiveness gap (that is, the difference between the biological or medicinal effect of the medicine itself on the body and its observed effectiveness in a particular population) by addressing issues such as acceptability of the treatment, compliance with regimes, attitudes towards the medicine etc., the reality is that in the majority of cases there is unlikely to be published qualitative evidence that could be synthesised that directly addresses the efficacy question. For example, while there might be substantial research into people’s lived experiences of particular illnesses, there is less likely to be evidence on people’s experiences of undergoing treatment A specifically. The most obvious exception to this is in long term conditions, or conditions where there is a notable impact on quality of life, where there is potentially substantial qualitative research – for example, cancer care or kidney dialysis. There is also a growing recognition within producers of clinical guidelines of the importance of qualitative evidence as a tool in implementation research because they “generate opportunities to examine complexity and include a diversity of perspectives” [20].

Arguably, QES could be more routinely useful in public health and social care topics where interventions tend to be more interpersonal or sociopsychological than biological and evaluations of views, perceptions and lived experiences (traditionally the domain of qualitative research) are more likely to be qualitative than in clinical medicine.

The line of argument about the likely availability of qualitative data is to a large extent borne out by the size of the QES that were carried out. With a modal number of four papers per QES they are, on average, relatively small. Themes from QES that contain so few studies may not score highly in a CERQual assessment (they are likely to be downgraded for adequacy unless the data from the studies is very rich), and this may restrict their usefulness as part of a decision-making process. Of the four large (> 50 papers) QES, two were part of the workplace health guideline [17], a non-clinical, public health guideline, and two were related to the attention deficit hyperactivity disorder: diagnosis and management guideline [16], which fits the model of a long-term condition with a notable impact on quality of life.

It is also plausible that the lack of relevant studies identified for most of the QES was due to either inappropriate research questions, or insufficient searching. Technical staff and information specialists producing QES within NICE are usually quantitative systematic reviewers and have little training in searching for or assessing qualitative evidence. Added to this, qualitative studies are notoriously poorly indexed in databases [21], qualitative study filters are still quite primitive in comparison to quantitative ones [21],[22], and qualitative literature searches are often quite specific (as opposed to sensitive) to limit the large amounts of irrelevant papers that need to be excluded during the sifting process.

The numbers of QES published per year does not appear to have the incremental increase that would be expected given the development of methods for QES over the 5 years in question, however this could be simply because the time period is too short to demonstrate any trend. It is also likely due to the varying patterns of NICE guideline publication. NICE guidelines take varying amounts of time to complete depending on a variety of factors, so there is not a consistent background rate of guideline publication against which the numbers of QES can easily be measured. The Tan paper however, reports that almost 50% of guidelines published in 2002–2007 ‘made use of qualitative studies’ (this is a slightly different measure to ‘undertaking a QES’ – the inclusion criterion for the current study. See below). During 2015–2019 that number was 28%, so a more detailed examination of the numbers over the lifetime of NICE could potentially reveal a year on year decrease in the number of guidelines using QES. A caveat here is that the Tan paper refers to ‘making use of qualitative studies’ but does not define this. There are guidelines from that period that report single or small numbers of qualitative studies but do not make any attempt at synthesis and therefore would not be considered for this study. The current content analysis only counted syntheses of two or more qualitative studies and did not count incidental use of single qualitative studies. This is likely to account for a good deal of the discrepancy.

### Purpose of QES undertaken

Almost half of the QES undertaken in 2015–2019 were carried out to address generic questions about barriers and facilitators to accessing a service or treatment, or about information needs relating to a condition. A substantial number of the remainder were about care and support needs of people with a specific condition. There seems in general little appetite to address more creative questions through QES even though the NICE manual [19] gives a broader list of examples than this including:

- What elements of care on the general ward are viewed as important by patients following their discharge from critical care areas?
- How does culture affect the need for and content of information and support for bottle or breastfeeding?
- What are the perceived risks and benefits of immunisation among parents, carers or young people? Is there a difference in perceived benefits and risks between groups whose children are partially immunised and those who have not been immunised?
- What information and support should be offered to children with atopic eczema and their families and carers?
- What are the views and experiences of health, social care and other practitioners about home-based intermediate care?

Occasional forays are made into more novel uses of QES. For example, in the Cataracts in adults: management guideline [18], a QES was undertaken to inform recommendations on wrong lens implant errors, specifically the questions “What are the procedural causes of wrong lens implant errors?” and “What strategies should be adopted to reduce the risk of wrong lens implant errors?”.

An avenue that does not seem to have been routinely explored by NICE is the use of QES as contextual grounding for guidelines. For example, a guideline about diabetes might usefully be underpinned as a whole by a QES that explored peoples experiences of living with, or caring for, people with diabetes, even though qualitative data to inform a QES about specific question within the guideline might not be available, the context would enable a guideline committee to frame their recommendation making in peoples lived experience of the condition.

### Quality of reporting

It is clear from Fig. 3 that there is good consistency within the ENTREQ criteria as to whether it is done well or poorly in NICE QES. Most criteria are either reported on by over 80 (out of 90) or by less than 45 QES. Very few criteria fall between these brackets.

Closer examination of the reporting criteria reveals that the criteria in the framework where the number of QES reporting the criterion are very high are all criteria that duplicate steps in quantitative systematic reviews and are therefore familiar to staff who are predominantly quantitative systematic reviewers. ENTREQ criteria relating to documenting the searching and sifting process, and to the creations of evidence tables of study characteristics are invariably done well, as is the presentation of the results of the methodological critical appraisal of the papers. Almost all of the criteria that duplicate steps in the quantitative systematic review process were reported in the QES (85 or more of the 90 QES).

Steps that are unique to QES, or where QES methods differ from quantitative systematic review methods, fare less well, and this is particularly the case with the criteria in the framework that require specific skills in methods for QES: data extraction, coding, use of software, and study comparison all fare poorly with less than 10% of the included QES reporting how (or if) they undertook these steps. Description of methods of qualitative synthesis also fared poorly with only around half of the QES reporting a synthesis approach in any detail.

### Variation over time and centre undertaking QES

The data presented here for different guideline producing centres are, at best, only indicative data. The picture they present of static guideline producing centres is potentially a misleading one. In the period under scrutiny (2015–2019), major changes were made to the way in which NICE contracts out work for guideline production. In the early stages of this time period, NICE had contracts with several external collaborating centres, mostly associated with academic units, and additionally an internal clinical guidelines team and a public health team. The external teams were responsible for specific areas of guideline production (for example, the National Collaborating Centre for Mental Health, or the National Collaborating Centre for Women’s and Children’s Health). The collaborating centres were replaced with two generic bodies, the National Guidelines Alliance and the National Guidelines Centre. These two bodies absorbed the functions, and in many cases the staff, of the Collaborating Centres. It is likely that the changing membership of review teams over that time has had an impact on the systematic review and QES processes that underpin the guidelines. [23].

In spite of this, there seem to be two general trends in the data contained in Figs. 4 and 5 that are important for this analysis. Firstly, that over time the quality of reporting of QES both overall, and by specific centres, has not improved in spite of clearer reporting frameworks and important methodological developments in QES. Secondly, the quality of reporting seems (in most cases) to be related to the centre producing the QES, with clear clusters of reviews of similar quality within centres. The exceptions, as discussed above, are the generic ‘contractor’ category and the public health team.

### Limitations

While we believe that the findings are robust, we acknowledge that the way that reviews are reported by NICE changed several times during the 5-year period under consideration. At various times multiple questions could be subsumed into single reviews or split across different review questions. This means that accurate counting becomes difficult, and some numbers are a near approximation based on counting and pragmatic decisions. Where numbers are uncertain this is reported.

The ENTREQ framework was not intended to be used for ‘scoring’ QES, and arguably not all ENTREQ reporting domains are equal in importance, nor was it designed as a formal reporting standard - its a general statement containing 21 items or criteria that can be broadly applied to common types of QES methodologies. As a framework, it is not well suited for more complex methodologies, however it is useful for simpler descriptive/aggregative methods as used in the QES described here.

The main purpose of this analysis was to better understand the quality of reporting of QES rather than why QES were or were not undertaken for specific guidelines. QES are relevant to a very specific range of research questions, and not all NICE guidelines could have benefitted from a QES. Further research would need to be undertaken to establish whether QES had been used appropriately in guideline development.

As with any documentary appraisal, it is unclear whether issues identified in this paper are due to the lack transparent reporting of the qualitative evidence syntheses or whether they relate to the conduct of the reviews themselves, or just to the reporting of them.

## Conclusion

Along with its international peers, including Cochrane and the World Health Organization (WHO), NICE is developing methods for the use of QES for producing health guidelines [6], [19]. To date this seems only to have been through relatively small numbers of QES, to address a very limited number of questions, primarily those about barriers and facilitators to service use and about people’s information and support needs when diagnosed with, or living with, a health condition. There is a potential to better understand the range of questions which qualitative evidence might be able to shed light on, and this in turn might make them more common as part of guideline production.

The focus of health guideline producing bodies on the use of systematic reviews of quantitative evidence and the relatively small amount of QES means that there is no noticeable improvement over time in the quality of QES produced. QES that are not produced by contractors who specialise in qualitative methods often lack transparent reporting of those aspects of the qualitative evidence synthesis that differ from the stages of a quantitative systematic review.

The clearest factor in the quality of a QES seems to be the team that undertook it. Teams which produce well-reported QES seem to do so consistently, and we can speculate that this may because they have staff with a particular interest or skill set in this area. Solutions to this might include ensuring that staff undertaking QES have appropriate skills and supervision, and providing clearer guidance about how a QES should be undertaken [6].

## Electronic supplementary material

Below is the link to the electronic supplementary material.

Supplementary Material 1

Supplementary Material 2

## Data Availability

The datasets used and analysed during the current study are included in Supplementary Material 2. The source documents are freely available on the NICE website (www.nice.org.uk).

## Footnotes

1 It is not possible to accurately count the number of review questions due to changes in the way that these are reported.
