# Supplementary material for: The conduct and reporting of qualitative evidence syntheses in health and social care guidelines: a content analysis
Source: BMC Med Res Methodol. 2022 Oct 12;22:267. doi: 10.1186/s12874-022-01743-1 (PMC9554851; doi:10.1186/s12874-022-01743-1)
Supplement: Supplementary file 1 — Supplementary Material 1 [file 12874_2022_1743_MOESM1_ESM.docx]

# **Additional File 1**

## **ENTREQ criteria, descriptions and coding rules**

Coding rules for specific ENTREQ criteria

| Item | Guide and description | Specific rules for this analysis. Criteria was marked as met if the QES did the following |
| --- | --- | --- |
| Aim | State the research question the synthesis addresses | Stated the review question to be addressed |
| Title & Synthesis methodology | Identify the synthesis methodology or theoretical framework which underpins the synthesis, and describe the rationale for choice of methodology (e.g. meta-ethnography, thematic synthesis, critical interpretive synthesis, grounded theory synthesis, realist synthesis, meta-aggregation, meta-study, framework synthesis). | Specified a methodology beyond noting that the methods set out in the NICE guideline manual had been followed or that ‘thematic synthesis was undertaken’. Detailed exposition of methods was not required. |
| Approach to searching | Indicate whether the search was pre-planned (comprehensive search strategies to seek all available studies) or iterative (to seek all available concepts until theoretical saturation is achieved). | No additional interpretation. |
| Inclusion criteria | Specify the inclusion/exclusion criteria (e.g. in terms of population, language, year limits, type of publication, study type). | No additional interpretation. |
| Data sources | Describe the information sources used (e.g. electronic databases (MEDLINE, EMBASE, CINAHL, PsychINFO, Econlit), grey literature databases (digital thesis, policy reports), relevant organisational websites, experts, information specialists, generic web searches (Google Scholar), hand searching, reference lists) and when the searches were conducted; provide the rationale for using the data sources. | No additional interpretation. |
| Electronic Search strategy | Describe the literature search (e.g. provide electronic search strategies with population terms, clinical or health topic terms, experiential or social phenomena related terms, filters for qualitative research and search limits). | No additional interpretation. |
| Study screening methods | Describe the process of study screening and sifting (e.g. title, abstract and full text review, number of independent reviewers who screened studies). | Description of process of sifting titles and abstracts then full text review sufficient for mark. |
| Study characteristics | Present the characteristics of the included studies (e.g. year of publication, country, population, number of participants, data collection, methodology, analysis, research questions). | If evidence tables were present in the review then this was considered to meet this criterion. |
| Study selection results | Identify the number of studies screened and provide reasons for study exclusion (e.g. for comprehensive searching, provide numbers of studies screened and reasons for exclusion indicated in a figure/flowchart; for iterative searching describe reasons for study exclusion and inclusion based on modifications to the research question and/or contribution to theory development). | Presence of a PRISMA style flowchart for study selection was considered sufficient for this criterion. |
| Rationale for appraisal | Describe the rationale and approach used to appraise the included studies or selected findings (e.g. assessment of conduct (validity and robustness), assessment of reporting (transparency), assessment of content and utility of the findings). | No additional interpretation. |
| Appraisal items | State the tools, frameworks and criteria used to appraise the studies or selected findings (e.g. Existing tools: CASP, QARI, COREQ; reviewer developed tools; describe the domains assessed: research team, study design, data analysis and interpretations, reporting). | Statement of critical appraisal tool used, or description of alternative critical appraisal process considered sufficient. |
| Appraisal process | Indicate whether the appraisal was conducted independently by more than one reviewer and if consensus was required. | No additional interpretation. |
| Appraisal results | Present results of the quality assessment and indicate which articles, if any, were weighted/excluded based on the assessment and give the rationale. | Presence of critical appraisal assessment in evidence table or summary of studies table considered sufficient. |
| Data extraction | Indicate which sections of the primary studies were analysed and how were the data extracted from the primary studies? (e.g. all text under the headings “results /conclusions” were extracted electronically and entered into a computer software). | No additional interpretation. |
| Software | State the computer software used, if any. | No additional interpretation. |
| Number of reviewers | Identify who was involved in coding and analysis. | No additional interpretation. |
| Coding | Describe the process for coding of data (e.g. line by line coding to search for concepts). | No additional interpretation. |
| Study comparison | Describe how were comparisons made within and across studies (e.g. subsequent studies were coded into pre-existing concepts, and new concepts were created when deemed necessary). | Presence of a logic model, theme map or similar was considered sufficient. |
| Derivation of themes | Explain whether the process of deriving the themes or constructs was inductive or deductive. | No additional interpretation. |
| Quotations | Provide quotations from the primary studies to illustrate themes/constructs, and identify whether the quotations were participant quotations or the author’s interpretation. | No additional interpretation. |
| Synthesis output | Present rich, compelling and useful results that go beyond a summary of the primary studies (e.g. new interpretation, models of evidence, conceptual models, analytical framework, development of a new theory or construct). | No additional interpretation. |

## **R code used to generate figures**

R code (RStudio version 1.3.1056) used to generate figures 6, 8 and 9. Other figures were generated in Microsoft Excel (Microsoft Office Professional Plus 2019).

Figures 1, 2 and 3 were produced in excel from the data spreadsheet using inbuilt excel functions.

**For figure 4 the following code was used on the ‘mean ENTREQ by centre/year’ supplementary spreadsheet**

library(ggplot2)

library(readxl)

#mean ENTREQ score by year across different centres

mean_entreq_by_year <- read_excel("mean entreq by year.xlsx",

+ col_types = c("character", "numeric", "numeric"))

View(mean_entreq_by_year)

df<- mean_entreq_by_year

ggplot( df, aes( x = Year, y = Mean ) ) +

geom_bar( stat = "identity" ) +

facet_wrap( ~ Centre ) +

ylab(“mean ENTREQ score out of 21”)

**For figure 5 the following code was used on the ‘clean data’ supplementary spreadsheet**

library(tidyverse)

df<-Clean_data_for_R_v_1_0

# plot of median ENTREQ score by authoring centre

At<- ggplot(df, aes(x=Author, y=Total))+

geom_boxplot()

At+

geom_jitter(shape=16, position=position_jitter(0.2))+

labs(x="Author of QES", y="Total ENTREQ score", title= "Median ENTREQ score by author of QES", subtitle= "Dots represent individual scores")

#End
